# The transfusion of prematures early school age follow-up (TOP 5): protocol for a longitudinal cohort study

**DOI:** 10.1186/s12887-025-05732-3

**Published:** 2025-05-15

**Authors:** Amy L. Conrad, Sara B. DeMauro, Haresh Kirpalani, Kristina Ziolkowski, Susan R. Hintz, Betty R. Vohr, Victoria Watson, Tarah T. Colaizy, Edward F. Bell, Jane E. Brumbaugh, Carla M. Bann, Sylvia M. Tan, Jamie E. Newman, Abhik Das

**Affiliations:** 1 University of Iowa, Iowa City, USA; 2 Children’s Hospital of Philadelphia, Philadelphia, USA; 3 University of Pennsylvania Perelman School of Medicine, Philadelphia, USA; 4 Stanford University School of Medicine, Stanford, USA; 5 Women & Infants Hospital of Rhode Island and Warren Alpert Medical School of Brown University, Providence, USA; 6 Mayo Clinic College of Medicine and Science, Rochester, USA; 7 Analytics Division, RTI International, Research Triangle Park, USA; 8 University of Iowa Carver College of Medicine, 146-B, CDD, 100 Hawkins Dr, Iowa City, IA 52242 USA

**Keywords:** Anemia, Child behavior, Child development, Transfusion, Neurodevelopment, Outcomes, Prematurity

## Abstract

**Background:**

Anemia of prematurity is a common concern for extremely low birth weight (ELBW) patients in the neonatal intensive care unit. The hemoglobin threshold at which the benefits of red blood cell transfusion outweigh the risks is unknown. The NICHD Neonatal Research Network Transfusion of Prematures (TOP) Trial evaluated whether higher (more liberal) hemoglobin transfusion thresholds resulted in improved survival without neurodevelopmental impairment at 22–26 months’ corrected age. A total of 1824 ELBW infants born at 22–28 weeks’ gestation were enrolled in the trial and randomized to either a restrictive or liberal set of red blood cell transfusion thresholds. Longer-term impacts of different transfusion thresholds in treatment for anemia of prematurity remain unknown. The Transfusion of Prematures Early School Age Follow-up (TOP 5) Study extends follow-up of all surviving children enrolled in the TOP Trial until early school age. It aims to assess longer-term cognitive and functional effects of differing transfusion thresholds in the newborn period for anemia in this large, multicenter cohort.

**Methods:**

Parents of surviving trial participants complete telephone questionnaires when their children are 3 and 4 years’ corrected age. A single in-person study visit takes place at early school age (5 years, 0 months to 7 years, 11 months’ corrected age). Children undergo a multidimensional assessment of functional outcomes, and parents complete a battery of questionnaires.

**Discussion:**

The TOP 5 Study will be the largest and most comprehensive evaluation to date of the functional early school age outcomes of children managed with different red blood cell transfusion thresholds during infancy for treatment of anemia of prematurity. This will substantially improve understanding of the longer-term neurological and functional outcomes of different transfusion thresholds; provide more refined evaluation of cognition, executive function, school readiness, motor skills, adaptive functioning, and behavior in former extremely preterm infants; and inform future clinical decision-making for treating anemia of prematurity.

**Trial registration:**

Clinicaltrials.gov ID: NCT01702805. Primary trial registration 10/05/2012; modified to include follow-up through school age 12/20/2018. This manuscript reflects version 3 of the trial protocol, dated 12/07/2020.

## Background

Anemia of prematurity is a common diagnosis among preterm infants in the neonatal intensive care unit (NICU), and its etiology is multifactorial. Extremely low birth weight (ELBW; with birth weight 1000 g or less) infants begin life with lower hemoglobin levels than full term infants and the subsequent months in the NICU involve frequent laboratory sampling as an essential aspect of intensive care, which results in further iatrogenic lowering of the hemoglobin level. The hemoglobin threshold at which the benefits of red blood cell transfusion outweigh the risks is unknown. The developing preterm brain may be susceptible to the effects of varying hemoglobin levels. A lower hemoglobin level, and thereby a lower oxygen carrying capacity, may place the developing brain at risk of hypoxic brain injury. Conversely, a higher hemoglobin level may place the developing brain at risk of injury from iron overload. There is also the possibility of hyperviscosity, or direct injury or inflammation induced by the transfused blood product.

### Iron load as a mechanism of insult in Anemia of prematurity and transfusion

In addition to oxygen transport, iron load is an important mechanism to evaluate in relation to early neural development. Iron is an essential nutrient for the developing brain. Iron is required for energy production and cellular metabolism. Iron status is measured clinically by erythropoiesis biomarkers, such as reticulocyte count, hemoglobin, and mean corpuscular volume. Iron maintains hemoglobin concentration when iron levels are low such that iron deficiency in the brain may exist before the peripheral blood reflects the deficiency [1]. Iron deficiency in the brain in the absence of anemia suggests that iron status in the brain and peripheral blood are not always concordant [2, 3]. In a neonatal lamb model, the brain iron concentration decreased relative to the iron content in red blood cells and relative to the reticulocyte count when the lamb was in negative iron balance [4]. Iron delivery to red blood cells appears to be preserved at the expense of brain iron. Thus, the iron status of the developing brain may not be reflected by peripheral blood measures.

Does a restrictive transfusion threshold risk the occurrence of iron deficiency at the neuronal level? With a restrictive transfusion threshold, infants experience a greater degree of anemia. This may place neonates at risk of the neurodevelopmental sequelae of iron deficiency. Later iron deficiency is associated with impaired processing speed, learning, and memory in children [5–8]. The adverse effects of iron deficiency depend on the timing and duration of the deficiency in relation to brain development. The developing hippocampus is particularly susceptible to iron deficiency. Iron is also involved in neurodevelopment through myelination and metabolism of neurotransmitters, such as dopamine. Tyrosine hydroxylase is an iron-containing enzyme involved in dopamine synthesis [9]. In addition, myelin formation depends on iron-dependent enzymes involved in fatty acid and cholesterol synthesis. Importantly, the effects of iron deficiency may endure well beyond infancy even after iron repletion [10–12].

Conversely, a liberal transfusion threshold may risk the occurrence of iron overload, another potential mechanism of brain injury in the neonate. Iron is involved in the formation of reactive oxygen species during cerebral reperfusion. In a rat model, deferoxamine, an iron chelator, reduced brain injury following cerebral hypoxia–ischemia [13]. Excess iron is an oxidant stress that is increasingly being evaluated for a potential role in age-related neurodegenerative disorders, such as Parkinson’s, Alzheimer’s, and Huntington’s diseases [14].

### Early morbidity and mortality of different transfusion thresholds

There is conflicting evidence regarding which hemoglobin thresholds for red blood cell transfusion should be used for the preterm population. The Prematures In Need of Transfusion (PINT) study and the Iowa transfusion trial along with the associated follow-up studies attempted to address the question of which hemoglobin thresholds to use for transfusion in the preterm population [15–20]. In the PINT study, there was no difference in head ultrasound findings between the restrictive and liberal threshold groups [16]. Follow-up at age 18–21 months for infants enrolled in the PINT study found no difference in the primary outcome of death or neurodevelopmental impairment for the restrictive (45.2%) versus liberal (38.5%) transfusion threshold groups (*p* = 0.09) [20]. When death and neurodevelopmental impairment were analyzed separately as secondary outcomes, statistical significance was not achieved although the directionality favored the liberal transfusion group for both outcomes. Post-hoc analysis revealed a difference in Mental Developmental Index (MDI) on the Bayley-II (4.3 points) favoring infants in the liberal transfusion threshold group relative to the restrictive transfusion threshold group (*p* = 0.030) [20]. Also, infants in the liberal group were less likely to have MDI < 85 (33.9% vs 44.9%, *p* = 0.016) [19]. In the Iowa transfusion trial, there was an increased incidence of significant brain injury (intraparenchymal brain hemorrhage or periventricular leukomalacia) in the restrictive transfusion group (12.2%) compared to the liberal transfusion group (0%) (*p* = 0.012) [15].

### The Transfusion of Prematures (TOP) Trial

**Fig. 1:**
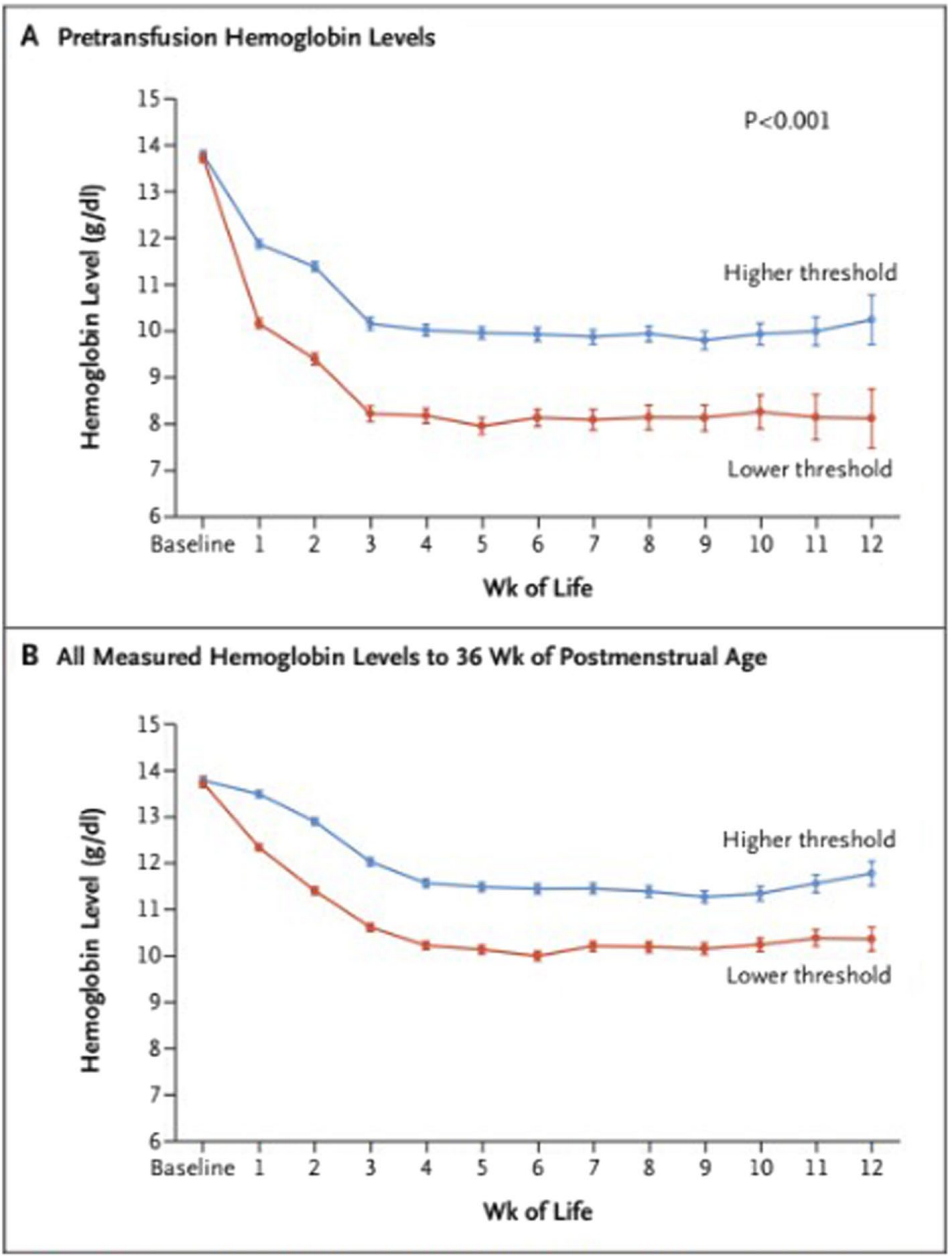
Separation of hemoglobin levels between the treatment groups. Hemoglobin levels in the higher-threshold and lower-threshold groups were recorded before enrollment and until 36 weeks of postmenstrual age. Values are means and 95% confidence intervals (indicated by I bars), adjusted for infant as a random effect. Hemoglobin tests were performed at clinical discretion and were not dictated by protocol. **A** shows the hemoglobin levels that prompted a red-cell transfusion. **B** shows all hemoglobin levels that were measured in the two groups during the treatment period. (Used with permission from NEJM)

Despite these prior, though inconclusive studies, the trend in clinical practice in recent decades has been toward lower, or more restrictive, hemoglobin transfusion thresholds. To evaluate the safety and efficacy of this practice, the National Institute of Child Health and Human Development (NICHD) Neonatal Research Network (NRN) completed the NHLBI-funded TOP Trial. The TOP Trial was a multicenter randomized controlled trial of ELBW infants born at 22–28 weeks’ gestation in which infants were randomized to either a restrictive or liberal set of red blood cell transfusion thresholds. The thresholds within each group varied based on chronological age and level of respiratory support. The primary aim of the TOP Trial was to determine whether higher, or more liberal, hemoglobin transfusion thresholds result in improved survival without neurodevelopmental impairment (NDI) at 22–26 months’ corrected age (CA). The trial intervention led to significant separation in hemoglobin levels in the two study groups between birth and 36 weeks’ postmenstrual age and significantly fewer transfusions through hospital discharge in the lower hemoglobin threshold group (Fig. 1). Of the 1824 infants enrolled, primary outcome data were available for 1692 (92.8%). Mortality, survival with severe complications, or serious adverse events at discharge from the hospital were not different between the higher- and lower-threshold groups. At follow-up (22–26 months’ CA), there continued to be no significant difference in rate of mortality or neurodevelopmental impairment [21].

### Lack of stability in neurodevelopmental measures

Measures of impairment obtained in toddlerhood are not always reliable predictors of functioning at school age. Cut off scores on cognitive measures obtained around 2 years of age can help identify those most likely to continue to perform significantly below average in school age. However, the positive predictive values and sensitivity are around 50% [22]. Hack and colleagues raised concern regarding the poor predictive validity of the Bayley at 20 months for cognitive function at school in the 2000 s [23]. Current research continues to show a lack of significant association between early Bayley scores and school performance [24].

This is highly relevant for clinical trials that evaluate outcomes of interventions conducted early in life. The Ment trial of indomethacin for the prevention of intraventricular hemorrhage (IVH), the Caffeine for Apnea of Prematurity Trial, the Victorian Infant Collaborative, and the Extremely Preterm Infants in Sweden Study (EXPRESS) all provide recent examples of trials involving preterm infants with conflicting neurocognitive outcome measures in toddlerhood and school age. From the Ment trial, growth in language scores was greater for those born very preterm compared to controls, reflecting catch-up with age [25]. Similarly, the Caffeine for Apnea of Prematurity Trial found a decrease in those identified as disabled between 18 months and 5 years of age [26]. In contrast, the Victorian Infant Collaborative found that only 46% of very preterm children remained in the same disability class between 2 and 8 years of age. There was a shift for those initially identified as not having a disability, to have a mild cognitive disability at 8 years of age [27]. The EXPRESS study also found an increase in rates of moderate to severe disability categorization from 2.5 to 6.5 years [28].

The reasons for differences between measures obtained in toddlerhood versus school age are multifaceted. Standardization of test administration for very young children is challenging, making reliable and valid assessment difficult. As the cognitive skills measured are still developing, there is a high degree of variability on performance [29], further limiting validity. Further, the provision of early screening and intervention may enhance outcomes while further insults or barriers to development may reduce outcomes. These limitations to early assessment are concerning because this is when routine clinical monitoring and clinical trial follow-up of extremely preterm children typically takes place. Fortunately, the stability of cognitive measures and predictive value for later functioning increase with development [30, 31], with higher stability of IQ across 4–12 years of age [32]. This makes long-term follow-up (e.g., school age) critical for clinical trials to fully understand the impact of early interventions on later outcomes.

### School-age outcomes of different transfusion treatment protocols

A subset (56%) of the original cohort in the Iowa transfusion trial was examined at school age (8–15 years). The restrictive transfusion group had a higher general ability index (GAI) on the Wechsler Intelligence Scale for Children, fourth edition (WISC-IV) than the liberal transfusion group (103.6 ± 15.7 vs 93.2 ± 0.1, *p* = 0.047) [17]. The children in the restrictive transfusion group similarly demonstrated better performance on reading ability than the children in the liberal transfusion group (105.8 ± 10.2 vs 93.9 ± 15.0, *p* = 0.002) using the Wide Range Achievement Test, third edition (WRAT-3). This signal favoring the restrictive transfusion threshold contrasted with earlier findings of the Iowa trial and the PINT study, which had suggested more favorable outcomes for the liberal transfusion threshold in infancy and toddlerhood.

A subset (*n* = 44) of the school age children from the Iowa transfusion trial also underwent brain MRI. Imaging revealed smaller intracranial volumes for children from the liberal transfusion threshold group compared to controls born at full term (*p* < 0.01), while there was no difference between the restrictive transfusion threshold group and controls [18]. Children in both the restrictive and liberal transfusion threshold groups had less cerebral white matter than control infants (*p* < 0.001). In contrast to previous reports of sex differences in children born premature where males were found to have worse outcomes [33, 34], the female children in the liberal transfusion threshold group demonstrated more abnormalities on brain imaging than the male children in the liberal transfusion threshold group [18]. A notable limitation of the school age follow-up was that a disproportionate number of females in the restrictive transfusion group were lost to follow-up.

The inconsistent relationships between transfusion threshold and outcomes in infancy and early childhood identified in earlier studies lead to important, unresolved questions. The primary aim of the TOP 5 study is to evaluate neurocognitive function in children born at ELBW who were managed with different hemoglobin transfusion thresholds as part of the TOP Trial. Given the differences in findings between the infancy, toddlerhood, and school age assessments in the earlier PINT and Iowa transfusion trials, it is crucial that we follow the infants enrolled in the TOP Trial to early school age to further evaluate neurocognitive outcomes dependent on transfusion threshold. As more children born at ELBW survive to adulthood, it is imperative that the standard of long-term follow-up move beyond toddlerhood. In addition to death and neurological functioning between transfusion thresholds, we will evaluate six domains at early school age in this large, diverse cohort: cognition, executive function, school readiness, motor skills, adaptive functioning, and behavior.

## Methods

### Study aims

The objective of the TOP 5 Study is to assess the early school age cognitive and functional effects of differing transfusion thresholds for management of anemia in the newborn ELBW infant.

The primary study aim is to characterize the neurological and functional outcomes of the ELBW population at 5–7 years’ CA based on neonatal transfusion thresholds.

Secondary study aims are to assess.

i) sex-specific effects on neurological and functional outcomes and
ii) transfusion threshold effects on school readiness, motor skills, memory, processing speed, behavior, and adaptive functioning.

### Design and setting

The TOP 5 Study is a longitudinal cohort study that follows all surviving participants in the TOP Trial [21], a multicenter randomized controlled trial of ELBW infants randomized to either a restrictive or liberal set of red blood cell transfusion thresholds. The thresholds within each group varied based on chronological age and level of respiratory support. The primary aim of the TOP Trial was to determine whether higher, or more liberal, hemoglobin transfusion thresholds result in improved survival without neurodevelopmental impairment (NDI) at 22–26 months’ CA. The TOP Trial completed recruitment in April 2017 with a cohort of 1824 infants enrolled across 19 academic centers in the NICHD Neonatal Research Network (United States, full list of study sites available at clinicaltrials.gov) [21].

**Fig. 2:**
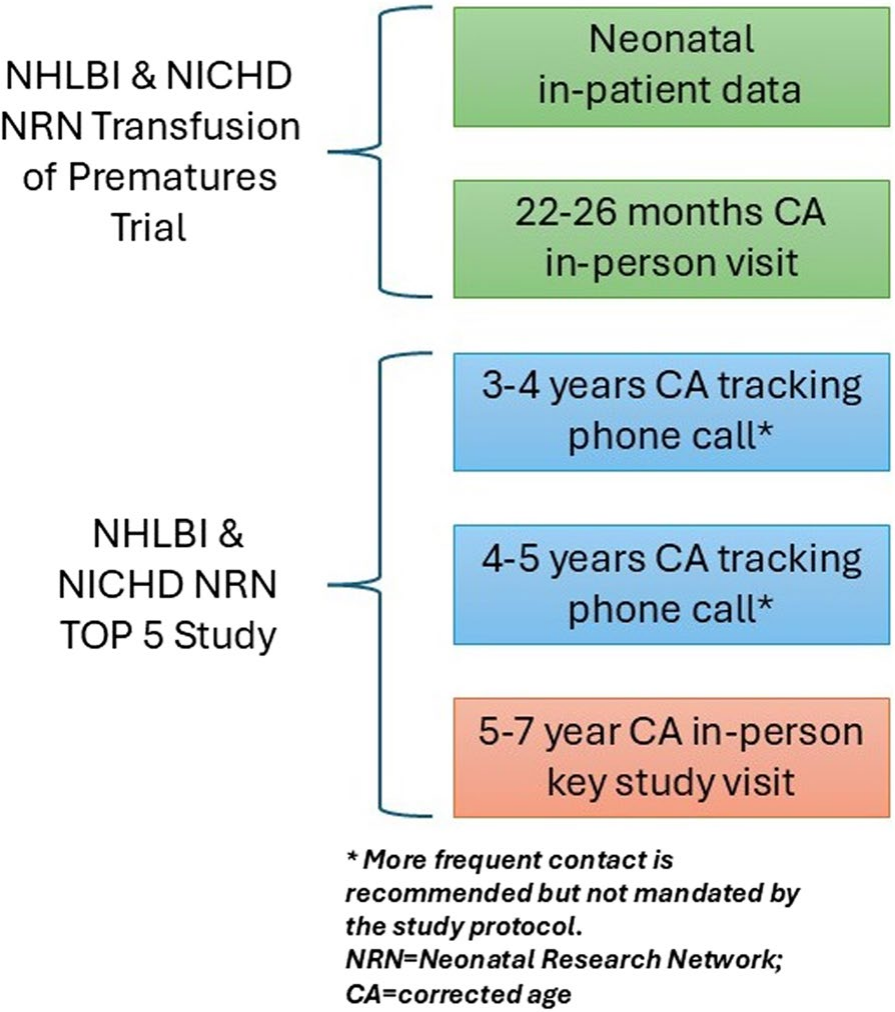
Timeline of study data collection and assessments

Following completion of the TOP protocol, families are contacted by phone when the children are 3 and 4 years’ CA. Then, as part of the TOP 5 Study, there is a final in-person visit when the child reaches early school age (Fig. 2). The school age visit had been planned for 5 years’ CA, with out of window testing permitted up to 7 years’ CA. However, due to recruitment delays caused by the COVID-19 pandemic, this window was extended up to (but not including) the date the child turns 8 years’ CA. Ages are adjusted for prematurity to avoid bias in cognitive scores [26, 35–37]. For the TOP Trial, it was determined to be impossible practically or ethically to blind either caregivers or parents to the assigned hemoglobin threshold. The open trial design allowed the clinicians to bypass the study algorithm in acute circumstances but then revert to the study algorithm following the protocol deviation. As such, the TOP Trial was an unblinded trial. However, examiners at the 22–26 month examination were blinded and examiners at the school age assessment are also blinded to exposure status.

In addition to the follow-up calls at 3 and 4 years’ CA, study sites maintain frequent contact with participating families until the final study visit is complete. This may include birthday cards, phone calls, emails or texts, and other approaches. Communication occurs at least every 6 months, with more frequent contact for families who are at high risk for loss to follow-up. During tracking phone calls when children are 3 and 4 years’ CA, families complete a questionnaire about recent illnesses, hospitalizations, and doctor visits.

The key, in-person, study visit is scheduled between 5 years’ CA and 7 years, 11 months’ CA. Attempts are made to schedule this visit after the child has entered kindergarten and at a time that is convenient for the family, e.g., in coordination with clinic visits or during weekends, holidays, or school vacations. Testing can be done in the clinic or at the family’s home. Families that reside over 200 miles from the study center are reimbursed for travel costs, or members of the study team travel to them to complete the assessment. If a child cannot be seen in-person for the key study visit, a lost to follow-up (LTFU) form is completed. If possible, questionnaires are completed by telephone with parent permission and results of school-based or other relevant assessments are submitted to the adjudication committee for review.

The protocol for the TOP 5 Study, including tracking and the single early school-age visit, was approved by each clinical center’s institutional review board (IRB). The NRN Research Participants Subcommittee reviewed the consent draft form prior to submission to the IRB. Written informed consent for the TOP 5 Study was obtained prospectively at the 2-year follow-up visit or is obtained in person at the key study visit, depending on local IRB permissions. Some participating center IRBs required assent for study visits by 7-year-old participants. The model consent form is available upon request from the Data Coordinating Center (DCC) at RTI International.

The TOP 5 Study is jointly overseen by a Clinical Coordinating Center (CCC) at the University of Iowa and a DCC at RTI. The TOP 5 Study Subcommittee includes members of the CCC and DCC (along with other members from the NRN) and meets quarterly to discuss study progress. When necessary, endpoints are adjudicated by members of the subcommittee with relevant expertise. The DCC manages all communications between the study subcommittee, sponsors, clinical sites, and other relevant parties.

### Participants

All surviving children enrolled in the TOP Trial are eligible for the TOP 5 Study. Parents provided written, informed consent for the TOP Trial. Inclusion criteria were: a) birth weight ≤ 1000 g, b) gestational age 22–28 weeks, and c) less than 48 h of age at the time of enrollment. Central randomization was performed within strata of birth weight (< 750 g versus 750–1000 g) and study center, with variable block sizes. Randomization was concealed to prevent allocation bias. The investigators of the TOP Trial designed the study using transfusion threshold triggers that fell within the current practice boundaries to assure buy-in of clinicians and developed the transfusion threshold algorithm by consensus.

### Measures

All study measures are listed in Table 1, along with estimated time for performing each measure. Measures were selected to provide harmonization across other NRN school-age studies. A brief summary of the different measures is provided below. For children who speak Spanish as a primary language, Spanish editions of the tests (when available) are administered by a Spanish language speaking clinician.

**Table 1:** Battery of assessments

Person Completing	Assessment Name	Measured Outcomes	Time (min)
**Child**	Gross Motor Function Classification System; Bimanual Fine Motor Function; neurologic exam (PE/VS)	Cerebral palsy	20
	Movement Assessment Battery for Children 2 (m-ABC 2)	Gross motor, fine motor function	35
	Differential Ability Scales-II (DAS-II)		67
	Core [Verbal Comprehension, Naming Vocabulary, Matrices, Picture, Similarities, Pattern Construction,Copying]	Cognition: verbal and nonverbal reasoning	
	Processing Speed [Rapid Naming, Speed of Information Processing]	Processing speed	
	Memory [Recall of Objects-Immediate, Recall of Digits Forward]	Memory: visual and verbal	
	School [Early Number Concepts, Matching Letter-Like Forms]	School readiness: colors, counting, letters, shapes, size/comparisons	
	Comprehensive Test of Phonological, Processing, 2nd Edition (CTOPP-2)	Phonological awareness: sound matching	5
	**Total Time**		**127**
**Parent**	Movement-ABC-2 checklist*	Developmental coordination disorder	10
	Adaptive Behavior Assessment System, 3rd Edition (ABAS-3)	Daily living skills and social skills subtests only	20
	Child Behavior Checklist (CBCL)	Adaptive and problem behaviors	15
	Behavior Rating Inventory of Executive Function, Preschool (BRIEF-P)	Executive function	15
	PedsQL	Quality of Life	10
	Social Communication Questionnaire (SCQ)	Social Functioning related to Autism Spectrum Disorders	10
	Medical history questionnaire	Medical History	10
	School, activities, and environment questionnaire	Social Risk	10
	**Total Time**		**100**

#### Training and certification

Initial training of study teams (coordinators, neurologic examiners, and psychologists) was completed during a two-day workshop (September 2017; Chicago, IL) in collaboration with training for the similarly designed HYBRiD Outcomes Study [38]. Each study psychologist then submitted a video of a full DAS-2 exam, CTOPP-2 Sound Matching Subtest, and self-critique for certification by the gold standard study examiners. For sites without a local Spanish language speaking examiner, certified bilingual DAS examiners traveled to conduct the assessment. One m-ABC examiner per site was also certified by sending a video and self-critique for review by a study gold standard examiner. Certified neurologic and m-ABC examiners were then permitted to certify additional examiners at their sites by reviewing materials, being observed, and then observing administration of these assessments. This is consistent with previously published NRN approaches to neurologic certification for the 2-year-old examination, which yields highly reliable results [39], and other NRN school age studies [38].

At the study midpoint (fall 2021), all DAS and m-ABC examiners underwent a pre-planned re-certification process. This was conducted via a virtual training workshop rather than a second cycle of video reviews due to the COVID-19 pandemic. The workshop was recorded for all examiners who could not attend or those who joined the study afterwards. All study source documents were reviewed centrally or by a second local person in order to monitor for both random and systematic administration or scoring errors [40].

#### Data and safety monitoring

The TOP 5 Study protocol is being carried out in accordance with Office for Human Research Protections (OHRP) and National Institutes of Health guidelines and requirements. Oversight is provided by the NRN Data Safety Monitoring Committee (DSMC), whose members include staff in neonatology, bioethics, maternal–fetal medicine, and biostatistics. The DSMC monitors safety data, enrollment, outcomes assessment, and attrition to ensure that the study will provide usable results with adequate statistical power. All communication with the DSMC funnels through the DCC. Prior to each planned (or ad hoc as necessary) meeting, the DCC reviews the study data, prepares interim reports, and arranges either an in-person meeting or teleconference to review these materials. The DCC also shares notification of DSMC recommendations to NRN Clinical Centers and NICHD/NHLBI. NICHD and NHLBI have the purview to act on DSMC recommendations to suspend or terminate the study should that become necessary for any reason. No specific stopping guidelines were planned a priori. The DSMC charter is available upon request from the DCC at RTI.

The TOP 5 quality management plan is maintained by the DCC. It outlines quality assurance procedures, specifications, audits, inspections, and other activities to ensure that the study meets applicable quality standards and regulatory requirements. The quality management plan was prepared in accordance with International Conference on Harmonization (ICH) Good Clinical Practice (GCP) E6 and applicable federal regulations for clinical studies. Both off-site (centralized) and on-site monitoring is performed by DCC and CCC personnel as needed.

Data are collected via a centralized web-based electronic data capture (EDC) system developed by RTI and hosted on FISMA-Low secure servers, ensuring all data are transmitted via Hypertext Transfer Protocol Secure (HTTPS). Access to the EDC system role-based, allowing each user to key in and view only data appropriate for their site location and trial responsibilities. The EDC includes real-time queries, such as warning triggers for out-of-range values, logic errors, data comparisons, and missing data. The system allows users to key in coded values as documented on the trial case report forms. The clinical data manager regularly reviews data and any unresolved queries.

Though anticipated to be infrequent, adverse events are monitored and reported per protocol guidelines. In the unlikely event of a serious adverse event (SAE) occurring at any of the clinical centers, it would be reported within 24 h of discovery to the NICHD Program Scientist (acting as medical monitor for all NRN studies), the DCC, and the DSMC as necessary (following established and codified processes). Local IRB policy for reporting SAEs would also be followed.

#### Neuro-developmental assessments

Select subtests from the *Differential Ability Scales-II* (DAS-II) [41] provide the primary assessment of cognitive skills and intelligence in the TOP 5 Study. The Global Conceptual Ability score of the DAS-II is strongly correlated with the full-scale intelligence quotient (IQ) score of the WISC-IV (correlation coefficient = 0.84) but is shorter to administer in this age range. The DAS-II also includes measures of school readiness, which are essential for assessment of functional outcomes. The DAS-II is increasingly used in school age follow-up studies of large neonatal cohorts [38, 42] and the Early Years Cognitive Battery is appropriate for children from age 3:6–6:11, with extended norms up to 7:11. This makes it ideal for testing our early school age participants who are at high risk for cognitive delay. Subtests related to school readiness, working memory, and processing speed are ideal for assessment of risk for school delay (a component of the primary outcome) and problems with executive function. The DAS-II has a supplementary Spanish-language version, which is administered to children whose primary language is Spanish.

The Sound-Matching subtest from the *Comprehensive Test of Phonological Processing, 2nd Edition* (CTOPP-2) [43] provides an additional measure of risk for school delay. Phonological awareness is a central early literacy skill and difficulties reflect high risk for later reading issues. A similar subtest from the *Test of Phonological Processing in Spanish* (TOPPS) [44] is administered to children whose primary language is Spanish.

For the neurologic exam, cerebral palsy is classified anatomically based on a hierarchical classification tree of cerebral palsy subtypes [45, 46] and functionally per *the Gross Motor Function Classification System* [47–49]. The *Movement Assessment Battery for Children, 2nd Edition* (m-ABC-2) [50] evaluates fine and gross motor coordination. Moderate motor coordination impairment is defined as a Total Impairment Score (TIS) between the 5 th and 14 th percentile and severe motor coordination impairment is defined as TIS < 5 th. The m-ABC is attempted for all children; however, in keeping with the diagnostic criteria for Developmental Coordination Disorder (DCD), abnormal motor coordination is only diagnosed in children who can participate fully in the m-ABC and do not have CP, blindness, or cognitive impairment (DAS-II Global Conceptual Ability < 70). The parent completes the m-ABC Checklist as a measure of the functional impact of a movement difficulty.

Difficulties with behavior (internalizing and externalizing problems) and attention are assessed with *the Child Behavior Checklist* (CBCL) [51]. This measure was used in this cohort at 22–26 months’ CA, enabling a longitudinal assessment of behavior and attention difficulties in the study population. We assess the presence of autism spectrum disorders with the Social Communication Questionnaire (SCQ) and quality of life with the *Pediatric Quality of Life* (PedsQL) inventory [52]. In addition to the working memory and processing speed subtests from the DAS, executive function is assessed the *Behavior Rating Inventory of Executive Function*. The preschool version (*BRIEF-P*) [53] is completed by parents of participants less than 7 years’ CA and the second edition is used for participants after they turn 7 years’ CA (*BRIEF-2*) [54]. Parental report of adaptive behavior (how the child uses their skills in everyday contexts) is assessed with *the Adaptive Behavior Assessment System, 3rd Edition* (*ABAS-3*) [55]. Additional parent questionnaires collect data about medical resource needs (hospitalization, medications, equipment needs, and therapy services), educational experiences and supports, screen time use, physical activity, and family impact.

#### Outcome definitions

The primary outcome at 5–7 years’ CA is a composite outcome of death or abnormal functional neurological outcome (defined as *any one* of the following):

1. Cognitive delay: General cognitive ability (IQ) > 2 SD below the mean (i.e., < 70) on the DAS-II
2. Motor delay: Cerebral palsy defined as Gross Motor Function Classification System Level 2 or higher or severe motor impairment measured as m-ABC Total Impairment Score < 5 th percentile
3. Lack of school readiness: Early school skills > 2 SD below the mean on the DAS-II number concepts and matching letter-like forms subtests (i.e., < 30)

The primary outcome will be a 3-level outcome variable (death, survival with functional impairment, and survival without functional impairment). Participants who were unable to complete cognitive testing due to cognitive/neurologicl impairment and were either diagnosed with autism or had an SCQ score above 15, were coded as having a cognitive delay. Participants who were lost to follow-up, but had information regarding cognitive, neurological, and academic functioning were discussed for adjudication of primary outcomes. Any confirmed delays were coded using the criteria above.

Secondary outcomes include the individual components of the primary outcome as well as additional measures of cognitive and functional development.

1. Death before assessment at 5 years
2. Death before assessment at 5 years or severe impairment (2 SD or more below the mean for cognition, motor, and/or school readiness)
3. Composite severe impairment (2 SD or more below the mean for composite measure of cognition, motor, and school readiness)
4. Cognition (below 1 SD of the mean for DAS-II: verbal comprehension, naming vocabulary, matrices, picture similarities, pattern construction, copying)
5. Moderate delay in motor skills (< 15.^th^ percentile for M-ABC-2 and/or GMFCS level II or greater)
6. School readiness (below 1 SD of the mean for DAS-II: number concepts, matching letterlike forms, CTOPP: sound matching)
7. Processing speed (below 1 SD of mean for DAS-II: rapid naming, speed of information processing)
8. Memory (below 1 SD of mean for DAS-II: recall of digits forward, recall of objects—immediate)
9. Gross motor coordination (< 15.^th^ percentile for M-ABC-2: total test score from manual dexterity, aiming and catching, static and dynamic balance; checklist motor score; checklist non-motor score)
10. Fine motor skills (Bimanual Fine Motor Function level II or greater)
11. Adaptive functioning (below 1 SD of mean for ABAS-3: general adaptive composite score)
12. Child Behavior Checklist (above 1 SD of mean for CBCL: total score)

### Analyses

#### Statistical analysis plan

To address the primary study aim, multinomial logit models will be conducted to compare the three-level outcome variable (death, survival with functional impairment, and survival without functional impairment) by study group (liberal versus restricted transfusion threshold), controlling for sociodemographic and medical characteristics, such as sex and birth weight, that have previously been shown to be related to the outcome, and for the clustering of participants within site. This model will allow testing of the hypothesis that children in the liberal transfusion group will have better functional outcomes. Contrast statements will be fit to compare (1) survival without functional impairment versus death/survival with functional impairment, and (2) survival without functional impairment versus survival with functional impairment. The impact of missingness will be assessed in separate sensitivity analyses.

Secondary outcomes will be compared among children who received transfusions based on restricted versus liberal thresholds using robust Poisson models for bi-level outcomes, and linear models for continuous outcomes, while controlling for similar covariates as for the primary analysis. Specifically, each individual component of functional impairment (cognition, motor skills, and skill readiness), as well as other outcomes of poor working memory, slow processing speed, behavior problems, and poor adaptive functioning will be compared for the restricted versus liberal transfusion groups. The differential impact of transfusion type (liberal versus restricted) by sex will be examined by additionally fitting models with a sex-by-transfusion group interaction term. If the test for interaction is statistically significant, separate models will be reported for boys and girls. All analyses will be two-sided and use an alpha level of 0.05. Analyses will be performed using SAS software v*9.4* (Cary, NC).

### Sample size and power estimates

Projected power for the TOP 5 Study composite primary outcome of functional impairment is based on estimated event rates from prior work within the NRN and from the neonatal literature. In the SUPPORT Neuro School Age study conducted by the NRN, 15% of surviving 6–7 year old former extremely preterm infants were diagnosed with moderate-to-severe cognitive impairment (IQ < 70) or cerebral palsy (GMFCS level 2–5) [56]. In a systematic review by Edwards et al., 25% of school aged children born with very low birth weight performed < 5 th percentile on the movement ABC [57]. A regional Australian cohort study reported “clinically important neurobehavioral impairment” (mild-to-major neurosensory, intellectual, educational, or behavioral impairment) in 55% of extremely low birth weight infants at 8 years [58]. Importantly, no study has assessed the composite outcome that is currently proposed for the TOP 5 Study. Based on these data, we hypothesize that at least one component of the functional impairment outcome (cognition, motor skills, and school readiness) will be present in about 40–50% of the school-age study cohort.

Of the original cohort of 1824 infants, we estimate a 15% death rate by the school age follow-up visit, and that 7% of survivors will have surpassed their follow-up windows before the study was funded, resulting in an initial sample of 1444 children. Of these, a 90% follow-up rate is anticipated, resulting in an estimated total of 1574 children (1300 followed + 274 deaths) with 787 in each transfusion threshold group having data on the primary outcome. Assuming a two-tailed test with p-value of 0.05 and centering proportions around 41% (45% for liberal transfusion and 37% for restricted transfusion), this sample size will provide 90% power to detect a difference of > 8% in survival without functional impairment (versus death/survival with impairment) between the two treatment groups. This difference corresponds to a small effect size using Cohen’s effect size measure h calculated as the difference of arcsine transformed proportions (h = 0.14).

We also assessed the power to evaluate sex differences in the impact of transfusion type based on the ratio of odds ratios for survival without functional impairment with liberal (versus restricted) transfusions among boys versus girls. We would have 84% power to detect a ratio of odds ratio of 1.35 or higher with a sample size of 1574, assuming an equal number of boys and girls and a *p*-value of 0.05.

## Discussion

The TOP 5 study has been designed to assess extremely preterm infants who were randomly assigned to different transfusion thresholds for treatment of anemia through the TOP Trial when they turn 5 years old. Findings from previous clinical trials (i.e., PINT & Iowa trials) were not conclusive but suggested that liberal thresholds (i.e., transfusing at higher hemoglobin levels) may be associated with lower rates neurodevelopmental impairment at follow-up around 2 years of age [15, 16]. The TOP Trial, designed to address to this uncertainty, did not find evidence supporting liberal thresholds conferring better mortality or neurodevelopmental outcomes [21]. However, outcomes measured as part of TOP were gross (i.e., death, brain injury, and cognition index) and conducted at too young of an age to assess more subtle and clinically meaningful group differences. Additionally, the school age follow-up of the Iowa transfusion trial suggested that liberal thresholds were associated with *lower* cognition index, academic achievement, and smaller intracranial volume [17, 18]. This contrasts with findings at 2 years of age.

The TOP 5 study extends this previous research with longer-term follow-up, permitting a comprehensive evaluation of neurological, cognitive, pre-academic, behavioral, and social functional outcomes. It is possible that risks related to lower oxygen carrying capacity and iron deficiency (anemia, lower thresholds) or iron overload (liberal transfusions) have a more subtle impact that are not measurable until later ages. Findings from the school-age follow-up of the Iowa transfusion trial found differences, favoring the restrictive group, in measures of verbal fluency, visual memory, and reading [17]. These are skills that cannot be evaluated until school age. Therefore, assessment at school entry with the comprehensive battery included within the TOP 5 Study protocol is essential to fully evaluate the potential impact of different transfusion thresholds on outcomes.

An additional novelty of the TOP 5 Study is the ability to evaluate potential sex effects in school age outcomes. The school age follow-up of the Iowa transfusion trial found that lower performance on verbal fluency was specific to females in the liberal transfusion group [59]. A series of further papers evaluated potential mechanisms behind these differences in ELBW infants at 12 months of age. Cytokine markers of inflammation were significantly higher after transfusion in females, and this was associated with lower cerebral white matter [60, 61]. Further, decreased pre-transfusion hemoglobin levels (anemia) were correlated with lower neonatal white matter in males, whereas increased hemoglobin was associated with worse performance on the Bayley-III in females [62]. Finally, 22–26 month outcome data from a cohort of TOP Trial participants found a similar pattern between pretransfusion hemoglobin and performance on the Bayley-III; lower hemoglobin was correlated with worse scores for males [63]. Together, these findings suggest that females may be particularly susceptible to inflammation related to transfusions while males are more susceptible to the effects of anemia. However, limitations with these studies – including unbalanced retention of sexes across both transfusion groups for the Iowa transfusion trial and lack of randomization to treatment groups for subsequent studies – leave uncertainty regarding the possible clinical relevance of the findings. The TOP 5 Study will extend evaluation of findings from Mostek [63], permitting a more refined evaluation of potential sex differences in longer term outcomes with a larger sample size that is balanced between treatment groups and sex.

Finally, similar to the HYBRiD Outcome Study [38], the use of “functional” measures provides a more patient- and family-centered evaluation of outcomes. Functional outcomes measure the integration of behaviors or skills that allow the child to achieve important everyday goals and are relevant in the context of everyday life. They are also required by the US Department of Education Office of Special Education Programs when conducting annual assessment of preschool (age 3–5 years) children with Individualized Education Programs (IEPs) [64]. The multidimensional construct of functional impairment that will be measured in the current study aligns with federal guidance about what data are relevant for understanding a child’s strengths, limitations, and needs during early childhood [64]. Importantly, this is the largest study to assess a comprehensive battery of school-age outcomes among ELBW infants randomly assigned to different red blood cell thresholds for treatment of anemia of prematurity. The tools are simple, feasible, and inexpensive. Therefore, results of this novel study can be easily generalized to future cohorts and can be used clinically. The results of our study may have important implications for public policy and resource planning for this vulnerable population of children.

In conclusion, it is critical to understand the effect of neonatal interventions such as red blood cell transfusion for anemia of prematurity at least through the time that its impact on important, child- and family-centered outcomes can be assessed. The TOP 5 Study will evaluate the trajectory of developmental outcomes in a high-risk cohort of former extremely preterm infants through early school age. The TOP 5 Study will determine if higher hemoglobin transfusion thresholds are associated with significant improvements in early school age functional developmental outcomes. Furthermore, this will provide a unique opportunity to evaluate potential sex differences in treatment protocols with a large trial cohort. The detailed data available from the TOP Trial, together with the TOP 5 Study, will help to fill these critical knowledge gaps. These data may be used to better counsel parents about possible outcomes for their children, to select high-risk children for earlier developmental interventions and therapies, and to inform the design of future research about the impact of anemia and transfusions on development.

## Data Availability

No datasets were generated or analysed during the current study.
